# Explorative and Exploitative Learning in Teams: Unpacking the Antecedents and Consequences

**DOI:** 10.3389/fpsyg.2020.02041

**Published:** 2020-08-14

**Authors:** Kai Zhao, Boqiang Zong, Lihua Zhang

**Affiliations:** Department of Human Resource Management, School of Labor and Human Resources, Renmin University of China, Beijing, China

**Keywords:** team explorative learning, team exploitative learning, leader power sharing, leader management control, team creativity, team task completion, overall team performance

## Abstract

Many organizations adopt team-based structures to better survive in the highly competitive environment. To achieve this goal, teams not only need to develop new ideas to adapt to the changing situations, but also follow standardized procedures to complete tasks effectively, suggesting the importance of ambidextrous capacity on addressing the paradoxical demands. However, we have little knowledge about both of how to respectively facilitate team ambidextrous learning (i.e., team explorative and exploitative learning) and how team the two learning activities contributes to team effectiveness. Using the multi-time and multi-source data gathered from 140 teams in 6 Chinese companies, we found that team leader’s power sharing and management control behaviors (i.e., ambidextrous leader behaviors) specifically enhanced team explorative and exploitative learning. In addition, our results showed that team explorative and exploitative learning drove overall team performance via the mechanisms of team creativity and task completion respectively. The theoretical and practical implications, limitations, and future research directions are discussed as well.

## Introduction

Under the trend of flat organizational structure, the use of teams in modern organizations is more and more popular, which spurs substantial research on factors which drive team performance (e.g., Gilson et al., 2005; Hülsheger et al., 2009; Mathieu et al., 2017). Similar as organizations, teams are encouraged to be creative and serve as the cornerstone of organizational innovation on the one hand; concurrently, teams also should be efficient in completing multiple tasks and act as the foundation of organizational daily operation on the other hand (Gilson et al., 2005). To satisfy the seemingly contradictory demands simultaneously, teams should be ambidextrous, which suggests that they should master both of exploration and exploitation (March, 1991). Building on team learning literature (Edmondson, 2002), scholars proposed the concept of team ambidextrous learning (defined as a set of team members’ collective learning behaviors including two types of activities: explorative and exploitative learning; Kostopoulos and Bozionelos, 2011) to capture such mastery. Here, explorative learning refers to the activities that facilitate a team to search, experiment with, and develop new knowledge, while exploitative learning depicts the activities that enable a team refine, recombine, and implement existing knowledge (Kostopoulos and Bozionelos, 2011).

Although existing studies have identified the antecedents of team ambidexterity as a unitary construct from team process or emergent state perspective (e.g., Jansen et al., 2016; Li et al., 2018), we have limited knowledge on how to specifically facilitate its two seemingly contradictory components (i.e., team explorative and exploitative learning). Because teams are faced with self-reinforcing tendencies and inherent tension between exploration and exploitation (March, 1991), specifying the respective antecedents of the two behavioral sets can enrich our knowledge on how to cultivate an ambidextrous team. Further, since exploration and exploitation drives performance through different routes (e.g., March, 1991; Lavie et al., 2010; O’Reilly and Tushman, 2013; Úbeda-García et al., 2018), differentiating the mediating mechanisms linking the two ambidextrous learning activities to overall team performance can unpack the black box of the effectiveness of team ambidextrous learning which is still an unsolved issue. The above two attempts are useful for researchers to better understand the nature of team explorative and exploitative learning, which are seemingly contradictory activities, but can coexist to contribute to team effectiveness. Specifically, we contribute to prior studies in three ways.

First, we extend theoretical understandings about the importance of leadership factors on boosting team explorative and exploitative learning. Leadership is regarded as a pivotal approach of enabling organizational contextual ambidexterity (Rosing et al., 2011; Tushman et al., 2011), because leaders can create a supportive context (e.g., discipline, stretch, support, and trust) to inspire individuals to make their own judgments about how to balance and satisfy the competing demands (Havermans et al., 2015; Luo et al., 2018). Drawing upon Rosing et al. (2011), ambidextrous leader behaviors are defined as s set of leading behaviors to foster exploration and exploitation in followers by increasing or reducing variance in their work behaviors, and they specified opening leading behaviors and closing leading behaviors to capture the two contradictory behavioral sets respectively. However, they just gave some behavioral “examples” for opening and closing leader behaviors (see Table 3 in Rosing et al., 2011 for details) rather than rigorously followed the procedures of scale development to generate and validate the items measuring the two types of leader behaviors. Thus, we don’t have mature scales to measure opening and closing leading behaviors. Because opening–closing leading behaviors share a fundamental similarity with the empower–control dimension mentioned in the construct of paradoxical leader behaviors (i.e., a homologous concept of ambidextrous leader behaviors) (Zhang et al., 2015), and that Chen et al. (2014) followed rigorous scale development steps to generate and validate the items measuring leader power sharing and management control, we adopted power sharing and management control to specifically represent variance increasing and variance reducing leader behaviors in this study. We argue that team leaders’ power sharing and management control behaviors nurture teams’ abilities to explore and exploit respectively. This attempt provides a new solution to cultivate team explorative and exploitative learning from the leadership perspective.

Second, our study justifies the effectiveness of team explorative and exploitative learning by respectively identifying the specific consequences (i.e., team creativity and team task completion) of team explorative and exploitative learning. Considering that explorative learning emphasizes exploring new possibilities in a creative way which shares a fundamental similarity with creativity, while exploitative learning highlights carrying out established task plans in a skilled and efficient way which is closely linked to following standardized procedures to complete task (Kostopoulos and Bozionelos, 2011), we propose that team explorative learning sparks team creativity, and that team exploitative learning contributes to promoting team task completion. By testing the two parallel linkages, we provide an evidence supporting the positive influences of the coexistence of ambidextrous activities at the team level. Besides, this attempt is helpful for researchers to differentiate the unique values of exploration and exploitation in facilitating team effectiveness.

Third, we extend research on the relationships between team explorative/exploitative learning and team performance by elaborating two different mechanisms (i.e., the change-oriented mechanism and the execution-oriented mechanism). In a dynamic environment, creativity and standardized procedures are two critical drivers for an effective team (Gilson et al., 2005). Because a creative team emphasizes challenging and changing the status quo (Shin and Zhou, 2007) and team explorative learning may enhance team creativity, we propose that team explorative learning facilitates overall team performance through the change-oriented mechanism. Since following standardized work procedures can promote the efficiency of the routine task completion, which contributes to team effectiveness (Gilson et al., 2005) and team exploitative learning may facilitate team task completion, we propose that team exploitative learning facilitates overall team performance through the execution-oriented mechanism. Our effort on justifying the two mechanisms contributes to an emerging body of literature on team-level ambidextrous hypothesis by unpacking the theoretical underpinnings of the relationship between team ambidextrous learning and team performance.

## Theory and Hypotheses Development

### Addressing Team Ambidextrous Learning

The concept of ambidexterity originates from the macro-management domain, which suggests that two contradictory yet interdependent elements constitute a pair of paradoxical relationship in an organization (e.g., Duncan, 1976; Andriopoulos and Lewis, 2009; García-Lillo et al., 2016). In order to manage the tensions embedded in these paradoxical relationships, organizations should be ambidextrous, implying that they can balance and integrate the two sides of paradoxes (Lewis, 2000; O’Reilly and Tushman, 2013; Luger et al., 2018; Knight and Cuganesan, 2020). From the learning perspective, March (1991) proposed that organizations should explore new knowledge while exploiting the existing knowledge to improve their adaptability to the environment. Exploration consists of the activities such as search, variation, risk taking, experimentation, and innovation, which is essentially a process of creating new knowledge, structures and procedures; exploitation includes the activities such as refinement, production, efficiency, selection, and implementation, which is essentially a process of improving and integrating existing knowledge, structures and procedures. They are seemingly contradictory, but complement with each other to promote organizational effectiveness (Solís-Molina et al., 2018).

Marco-management scholars have identified three approaches to address the tension between exploration and exploitation: (1) structural separation that argues for separate structures within the same organization to accommodate the opposing competencies of exploration and exploitation (e.g., Tushman and O’Reilly, 1996); (2) temporal balancing that defined as sequential switches between exploration and exploitation by an organization (e.g., O’Reilly and Tushman, 2013); (3) contextual approach that emphasizes the capacity of behavioral integration that pursues both exploration and exploitation (e.g., Gibson and Birkinshaw, 2004). Research focus of the approach to addressing ambidexterity shifts from structural to contextual ambidexterity, which suggests that exploration and exploitation are not only distinct activities, but also can coexist concurrently within an organizational unit (Gibson and Birkinshaw, 2004; Lavie et al., 2010).

Recently, an increasing number of scholars in the field of micro-management pay more attention to ambidextrous phenomena at the team level. Team is a closely social interaction system (Mcgrath et al., 2000; Cronin et al., 2011; Mathieu et al., 2017); thus, it is unlikely to be divided into two separated sub-units (i.e., exploratory unit and exploitative unit). Further, as team is a relative independent work unit that needs concurrently adapt to the changing environment and complete routine task effectively in daily operation (Kostopoulos and Bozionelos, 2011; Jansen et al., 2016; Li et al., 2018), it is also less likely for a team to adopt a temporal approach to address ambidexterity. Therefore, we argue that compared with structural and temporal approach, the contextual approach is more feasible for a team to simultaneously pursue explorative and exploitative goals.

### Ambidextrous Leader Behaviors and Team Ambidextrous Learning

Drawing on the loose–tight principle mentioned in previous paradox literature (Sagie, 1997), we focus on team leader’s empowering (i.e., power sharing) versus controlling (i.e., management control) behaviors as the specific ambidextrous leader behaviors in this study. A recent empirical study also identified “control and empowerment” as a critical component of paradoxical leader behaviors (i.e., a homologous concept of ambidextrous leader behaviors) and demonstrated its predictive validity to both of employees’ adaptive (i.e., exploration-type) and proficient (i.e., exploitation-type) behaviors (Zhang et al., 2015). Power sharing refers to the supervisor’s leading behaviors that delegates authority to followers to do their tasks with more autonomy and allows followers to participate in the team-level decision-making processes; whereas management control is defined as the leadership behaviors that facilitates control over subordinates through setting clear work goals for followers and monitoring their work progresses to ensure team performance (Kirkman and Rosen, 1999; Chen et al., 2014). Similar as the relationship between exploration and exploitation, power sharing and management control are seemingly incompatible; however, based on the empowerment literature, they can coexist and complement with each other to improve team performance. As Arnold et al. (2000) stated, empowered team is semi-autonomous—there is no thorough authorization, and proper control is necessary to ensure the effectiveness. If a supervisor empowers the subordinates without any management control may increase the subordinates’ ambiguity and uncertainty in decision making and responsibility for delivering outcomes (Chen et al., 2014), causing them to struggle in the repetitive process of trial and error. Accordingly, in order to overcome the weaknesses of power sharing and enhance team exploration and exploitation simultaneously, on the one hand, team leaders should share power with subordinates to enable them to explore new possibilities, and on the other hand, they should also set a clear task goal and monitor team processes (i.e., control-related leading behaviors) to promote usefulness and efficiency of the subordinates’ work behaviors.

Specifically, we expect that leader power sharing is positively related to team explorative learning. The essence of exploration is the increase of variance (March, 1991). Thus, to enhance team explorative learning, team leader needs to shape a discretionary team context that fosters variance of team members’ behaviors. Leader power sharing may be useful to cultivate such context because it emphasizes leader’s authority delegation and members’ participative decision making (Chen et al., 2014). In this regard, team members have more autonomy to conduct their tasks based on their own decisions, which increases variance in a team. Then, the variance triggers more explorative activities, such as exploring new knowledge, experimenting with new ideas, and taking risks. Existing studies found that leader’s empowering-oriented behaviors can promote their followers to search new information with respect to work, learn new knowledge and skills, and develop new solutions to solve unfamiliar problems (e.g., Burpitt and Bigoness, 1997; Zhang and Bartol, 2010), all of which are related to explorative activities. Therefore,

Hypothesis 1a. Leader power sharing is positively related to team explorative learning.

In contrast, we argue that leader management control is positively related to team exploitative learning. The core idea of exploitation is the reduction of variance (March, 1991; Gupta et al., 2006). Thus, to promote team exploitative learning, it’s necessary for team leaders to build a relative constrained context to reduce variance of team members’ behaviors. Leader management control may be effective to shape such context because it focuses on setting up performance standards and monitoring team processes (Chen et al., 2014). Both of the two behavioral sets lead to team members’ rule-following behaviors, which are closely bounded up with variance reduction. In the context that encourages reducing variance, team members are more likely to engage in exploitative activities, such as refining existing knowledge and skills, adopting well-structured approaches to solve problems, and implementing established plans effectively. Supporting this argument, existing studies confirmed that leaders exhibiting controlling-oriented behaviors can foster their followers to focus on efficiency, integrate extant knowledge to solve problems, and refine existing work procedures (e.g., Lewis et al., 2002), all of which are associated with exploitative activities. Thus,

Hypothesis 1b. Leader management control is positively related to team exploitative learning.

### Team Ambidextrous Learning and Team Effectiveness

We first speculate that team explorative learning predicts team creativity. Team creativity reflects to what extent the members come up with novel and useful ideas to address the unexpected problems in teamwork (Shin and Zhou, 2007). Team creativity is not a simple aggregation of team members’ individual creativity, but in need of intragroup creative-relevant processes including the collective activities such as elevating new goals, eliciting and appreciating different viewpoints, providing feedbacks, and coordinating contributions (Taggar, 2002). Based on the above definition of team explorative learning, it can be regarded as a variance-oriented intragroup learning process, which encourages team members to challenge existing goals, take risks to experiment different novel methods, and activate their creative thinking to solve problems (Kostopoulos and Bozionelos, 2011; Jansen et al., 2016). These variance-oriented collective behaviors can be essentially classified into team creative-relevant processes, which thereby facilitates the generation of creative ideas and opinions in a team. Thus,

Hypothesis 2a. Team explorative learning is positively related to team creativity.

Then, we propose that team exploitative learning promotes team task completion. Team task completion refers to the extent to which the focal team meets the preset goals and how well its output fulfills the team’s mission (Hackman, 1987; Zellmer-Bruhn and Gibson, 2006). Because a team’s mission and goals are usually clear-defined and operationalized in organizational routines, team members need develop standardized work procedures and follow them to conduct the established mission and goals efficiently. According to the above definition of team exploitative learning, it can be regarded as an efficiency-oriented intragroup learning process, which enables team members to integrate existing knowledge to establish a unified framework, follow routines to do tasks, and adopt the “best practices” to solve problems efficiently (Kostopoulos and Bozionelos, 2011; Jansen et al., 2016). These efficiency-oriented collective behaviors are in favor of both of the formation and the adoption of standardized work procedures, which thereby promotes the completions of preset tasks in a team. Therefore,

Hypothesis 2b. Team exploitative learning is positively related to team task completion.

Because environment is becoming more and more dynamic and competitive, organizational teams always face non-routine and multifaceted tasks, suggesting that the connotation of team effectiveness is beyond the single dimension of task performance. At the operationalization level, we use the term of “overall team performance” to tap team effectiveness in this study (Ancona and Caldwell, 1992; Van Der Vegt and Bunderson, 2005). This variable covers multiple criterions (e.g., efficiency, quality, and productivity) which are suitable for evaluating different team outcomes. Achieving overall team performance not only refers to the completion of routine tasks through adhering to established procedures and objectives, but also emphasizes the ability of handling non-routine work via creating new knowledge (Gilson et al., 2005). The former is in need of executive forces, which benefits from exploitative learning (March, 1991) and manifests as high levels of task completion (namely as the execution-oriented mechanism to drive performance); the latter requires change forces, which gets profit from explorative learning (March, 1991) and is indicated by high levels of creativity (namely as the change-oriented mechanism to enhance performance).

For the change-oriented mechanism, we focus on the mediating role of team creativity. We argue that team creativity promotes the overall effectiveness of a team. This is because teams which adopt novel and useful ideas are more likely to achieve the radical breakthroughs, as well as continual optimization in team operation processes, both of which are critical drivers of team performance (Gilson et al., 2005; Hülsheger et al., 2009). Additionally, creative teams usually have a participative climate, which enables their members to engage in plentiful social interactions with both of their teammates and cross-team colleagues (Anderson and West, 1998). These social activities not only produce fresh and diverse domain knowledge, but also facilitate intra- and inter-team cooperation, both of which are helpful to improve overall team performance (Drach-Zahavy and Somech, 2001; Perry-Smith and Shalley, 2003; De Vries et al., 2016; Srikanth et al., 2016). Taking the positive association between team explorative learning and team creativity into account, we propose,

Hypothesis 3a. Team creativity mediates the positive relationship between team explorative learning and overall team performance.

For the execution-oriented mechanism, we mainly elaborate the mediating role of team task completion. We suggest that team task completion is also positively associated with the overall effectiveness of a team. Team task completion reflects to what extent a team can achieve its established goals efficiently (Hackman, 1987). In this regard, a team with high levels of task completion can handle and solve the routine problems timely, which is a critical indicator of its overall effectiveness (e.g., Ancona and Caldwell, 1992; Gilson et al., 2005; Hu and Liden, 2015). In addition, a team with high levels of task completion signals that this team is reliable, efficient and effective, which may promote its internal cohesion among team members (e.g., Mullen and Copper, 1994; Chang and Bordia, 2001; Mathieu et al., 2015) and external reputation to relevant stakeholders (e.g., Tyran and Gibson, 2008; Peralta et al., 2015, 2018). Both of these two elements are key components of a team’s overall effectiveness. Integrating the positive relationship between team exploitative learning and team task completion and the above arguments, we expect,

Hypothesis 3b. Team task completion mediates the positive relationship between team exploitative learning and overall team performance.

**FIGURE 1:**
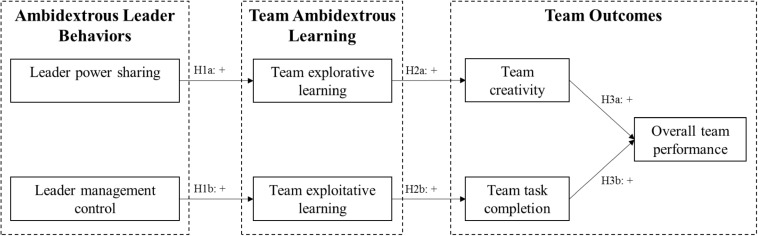
Hypothesized model.

Figure 1 summarizes all the hypotheses and depicts our theoretical framework.

## Materials and Methods

### Participants and Procedures

Data were collected from 140 teams in six technology-based companies located in China. With the assistance of company HR managers, all the voluntary participants completed their questionnaires in their companies’ conference rooms under the guidance of the researchers. After finishing the questionnaires, the researchers sealed them in an envelope, and then brought them back. We collected data from three sources (team members, team leaders, and top executives) at three time points. At Time 1, team members were asked to rate their perceived direct supervisor’s ambidextrous leader behaviors (i.e., power sharing and management control), and demographic variables. The questionnaires were distributed to 884 members from 140 teams. Then, 783 valid questionnaires from 132 teams were returned, reflecting response rates of 88.57% at the individual level, and 94.29% at the team level. Three months later (Time 2), the 783 members who finished the first round of survey were invited to rate their perceived team ambidextrous learning (i.e., team explorative and team exploitative learning); in addition, leaders of the 132 teams were asked to rate their team’s creativity and task completion, and their demographic information. Then, 774 valid questionnaires from 130 teams were returned, suggesting response rates of 98.85% at the individual level, and 98.48% at the team level. Another 3 months later (Time 3), CEOs from the six companies were invited to rate the overall performance of the participated teams from their companies (i.e., the CEO of a biotechnology company in Beijing evaluated the overall performance of 24 teams; the CEO of another communication technology company in Beijing evaluated the overall performance of 15 teams; the CEO of a software development company in Nanjing evaluated the overall performance of 29 teams; the CEO of a automation equipment manufacturing company in Suzhou evaluated the overall performance of 20 teams; the CEO of a communication technology company in Qingdao evaluated the overall performance of 28 teams; the CEO of an agricultural technology company in Taiyuan evaluated the overall performance of 14 teams). Finally, all the CEOs’ questionnaires (130 in total) were returned. In our final sample of 774 individuals from 130 teams, 73.23% of the members were male, the average team tenure was 3.65 years, the average team size was 9.34 individuals, and 75.70% of the members held a bachelor degree or above.

### Measures

All the variables in this study were measured by the established scales. Unless otherwise specified, we adopted a six-point Likert-type scale (1 strongly disagree to 6 strongly agree) for all measures. To ensure the reliability and validity of the final scales, all the items were first translated into Chinese by one bilingual scholar and then translated back into English by another, thereby to a high degree of clarity and accuracy (Brislin, 1986). For complete scale information, please refer to Appendix 1.

#### Ambidextrous Leader Behaviors

At Time 1, we measured team leaders’ power sharing and management control using the scales adapted from Chen et al. (2014). The scale of leader power sharing has seven items. All the items are as follows: “My supervisor does not interfere with work that is within my job responsibility,” “My supervisor fully delegates and lets me take full charge of my job,” “My supervisor gives me authority to make autonomous decisions in my job,” “When there is a problem at work, my supervisor listens to my ideas and suggestions,” “My supervisor often provides me opportunities to express my ideas,” “My supervisor asks for my views before making decisions concerning me and my job,” and “When making decisions, my supervisor respects and values my suggestions.” The Cronbach’s alpha for this scale was 0.89. Leader management control was measured by seven items. All the items are as follows: “My supervisor sets work goal for me and requires me to ensure its achievement,” “My supervisor emphasizes work goal,” “My supervisor periodically checks if I am accomplishing my work goals,” “My supervisor emphasizes work outcome,” “My supervisor would seriously point out my work mistakes,” “My supervisor often asks me about my work progress,” and “My supervisor periodically examines whether my work is going on smoothly.” The Cronbach’s alpha for this scale was 0.86.

#### Team Ambidextrous Learning

At Time 2, we measured team explorative and exploitative learning using the scales adapted from Kostopoulos and Bozionelos (2011). Five items were used to measure team explorative learning. All the items are as follows: “Team members were systematically searching for new possibilities during the project,” “Team members offered new ideas and solutions to complicated problems,” “Team members experimented with new and creative ways for accomplishing work,” “Team members evaluated diverse options regarding the course of the project,” and “The members of our team developed many new skills during the project.” The Cronbach’s alpha for this scale was 0.89. Team exploitative learning was measured by five items. All the items are as follows: “The members of our team recombined existing knowledge for accomplishing work,” “In our team, we primarily performed routine activities,” “During the project, our team implemented standardized methodologies and regular work practices,” “Team members improved and refined their existing knowledge and expertise during the project,” and “Team members mainly used their current knowledge and skills for performing their tasks.” The Cronbach’s alpha for this scale was 0.81.

#### Team Creativity

At Time 2, team creativity was measured by a four-item scale (1 very poorly to 6 very much) developed by Shin and Zhou (2007). All the items are as follows: “How well does your team produce new ideas?” “How useful are those ideas?” “How creative do you consider your team to be?” and “How significant are those ideas to your organization?” Items used a six-point frequency scale ranging from “seldom” to “always.” The Cronbach’s alpha for this scale was 0.87.

#### Team Task Completion

At Time 2, team task completion was measured by a five-item scale developed by Zellmer-Bruhn and Gibson (2006). All the items are as follows: “This team achieves its goals,” “This team accomplishes its objective,” “This team meets the requirements set for it,” “This team fulfills its mission,” and “This team serves the purpose it is intended to serve.” The Cronbach’s alpha for this measure was 0.92.

#### Overall Team Performance

At Time 3, overall team performance was measured by a five-item scale (1 far below average to 6 far above average) developed by Van Der Vegt and Bunderson (2005). The specific performance criteria include efficiency, quality, overall achievement, productivity, and mission fulfillment. Top executives were asked to rate the focal team’s performance relative to other teams that performed similar tasks (1 far below average to 6 far above average). The Cronbach’s alpha for this measure was 0.91.

#### Control Variables

Following existing studies (Shin and Zhou, 2007; Tsai et al., 2012), we controlled for team tenure and team size to partial out their influences on team outcomes. In addition, given the potential confounding effects of team functions on the hypothesized relationships, we coded team functions as the following six dummy variables: R&D, manufacture/operations, marketing and sales, customer service, functional management, and operation support.

#### Data Aggregation

Because all of our focal variables were at the team level, we empirically checked the appropriateness of aggregating the responses of individual team members to the team level. First, we calculated the Rwg indexes for a rectangular (uniform) null distribution to assess the interrater agreement (James et al., 1984). The mean Rwg values were 0.84 for leader power sharing, 0.87 for leader management control, 0.82 for team explorative learning, 0.88 for team exploitative learning, all of which were above the conventional cutoff value of 0.70 (James et al., 1984). Further, following the suggestion of Biemann et al. (2012), we also computed the RWG for alternative null distributions (i.e., a slight skew distribution, a moderate skew distribution, and a normal distribution), because the actual distribution of our measures may be not uniform. The detailed results were reported in Appendix 2. The RWG values derived from a rectangular or uniform distribution should be viewed as an upper limit; the RWG values based on the alternative null distributions (termed as “measure-specific” in Appendix 2) can be interpreted as a theoretical lower bound of within-group agreement. Second, based on the one-way analysis of variance (ANOVA), we obtained the following acceptable ICC values: power sharing (ICC1 = 0.25, *F* = 1.74, *p* < 0.001; ICC2 = 0.70); management control (ICC1 = 0.22, *F* = 1.45, *p* < 0.01; ICC2 = 0.66); team explorative learning (ICC1 = 0.31, *F* = 2.14, *p* < 0.001; ICC2 = 0.76); and team exploitative learning (ICC1 = 0.42, *F* = 2.79, *p* < 0.001; ICC2 = 0.83). These values are all in the conventional value ranges of ICC1 and ICC2 of the aggregated team-level variables in the organizational literature (e.g., Bliese, 2000; Liao and Chuang, 2007). Therefore, we justified the data aggregation of our focal team-level variables.

### Analyses

As top executives rated multiple teams’ overall performance, we adopted the multilevel liner model to control the rater effect (Bliese, 2002; Raudenbush et al., 2011). Thus, we used the methodology of Multilevel Path Modelling (MPM) to test the overall model as a whole (Zhang et al., 2009) via Mplus 7.4. Because our data was from six companies and all the focal variables were operationalized at the team level, we group-mean-centered all the variables except for overall team performance to reduce the interference of companies’ influences on the results. In addition, when examining the mediation effects, we used R program to conduct the Monte Carlo simulation with 5000 replications (Preacher and Selig, 2012) to construct the 95% confidence intervals of the indirect effects, which has been widely used when testing the multilevel mediation effect (e.g., Lanaj et al., 2014; Huang et al., 2016).

### Construct Validity

Using Mplus 7.4, we conducted two sets of CFA (i.e., one for the variables based on the report of team members and one for the variables based on the report of team and organizational leaders) to assess the discriminant validity of the focal variables. For the variables rated by team members (i.e., powering sharing, management control, team explorative learning, and team exploitative learning), the four-factor model had an acceptable fit index (χ*^2^* = 1354.17, *df* = 246, χ^2^*/df* = 5.50, CFI = 0.89, TLI = 0.87, RMSEA = 0.08), and fitted the data better than the two-factor model in which leader powering sharing and management control were combined as a factor, and team explorative and exploitative learning were combined as another factor (χ*^2^* = 3080.06, *df* = 251, χ*^2^/df* = 12.27, CFI = 0.71, TLI = 0.68, RMSEA = 0.12) and the one-factor model in which all the variables were combined as a single factor (χ*^2^* = 5369.47, *df* = 252, χ^2^*/df* = 21.31, CFI = 0.47, TLI = 0.42, RMSEA = 0.16). For the variables rated by team and organizational leaders (i.e., team creativity, team task completion, and overall team performance), the three-factor model offered a reasonable fit indexes (χ*^2^* = 105.46, *df* = 74, χ*^2^/df* = 1.43, CFI = 0.97, TLI = 0.97, RMSEA = 0.06), and fitted the data better than the two-factor model in which team creativity and team task completion were regarded as a factor and overall team performance as another factor (χ*^2^* = 1322.60, df = 91, χ*^2^/df* = 14.53, CFI = 0.79, TLI = 0.75, RMSEA = 0.16) and the one-factor model in which all the variables were combined as a single factor (χ*^2^* = 657.34, *df* = 77, χ*^2^/df* = 8.54, CFI = 0.53, TLI = 0.44, RMSEA = 0.24). Overall, the above results provide support for the discriminant validity of our measures.

## Results

Table 1 showed the descriptive statistics and the correlations among our focal variables.

**TABLE 1:** Descriptive statistics and correlations among study variables.

**Variables**	**Mean**	***SD***	**1**	**2**	**3**	**4**	**5**	**6**	**7**	**8**	**9**	**10**	**11**	**12**	**13**	**14**	**15**
(1) Team tenure	3.62	4.05															
(2) Team size	9.15	5.92	0.21*														
(3) R&D	0.63	0.48	−0.19*	0.04													
(4) Manufacture/operations	0.13	0.34	0.21*	0.02	−0.52**												
(5) Marketing and sales	0.12	0.33	–0.10	0.03	−0.49**	–0.15											
(6) Customer service	0.02	0.15	–0.09	0.05	−0.20*	–0.06	–0.06										
(7) Functional management	0.04	0.19	0.00	–0.05	−0.27**	–0.08	–0.08	–0.03									
(8) Operation support	0.02	0.15	0.24**	–0.03	−0.20*	–0.06	–0.06	–0.02	–0.03								
(9) Leader power sharing	4.86	0.39	–0.03	–0.12	0.09	–0.13	–0.09	0.08	0.06	–0.04	(0.89)						
(10) Leader management control	4.98	0.34	0.12	–0.12	0.11	0.16	–0.03	0.11	0.03	0.04	0.31**	(0.86)					
(11) Team explorative learning	4.63	0.36	−0.17*	–0.03	0.14	–0.11	0.03	0.06	0.04	–0.16	0.39**	0.24**	(0.89)				
(12) Team exploitative learning	4.80	0.32	0.09	–0.12	–0.02	0.05	–0.11	–0.04	0.09	0.07	0.31**	0.30**	0.42**	(0.81)			
(13) Team creativity	4.18	0.87	−0.19*	–0.06	0.24**	–0.13	–0.03	–0.08	−0.19*	0.06	0.22*	0.16	0.33**	0.13	(0.87)		
(14) Team task completion	4.94	0.72	0.00	–0.04	0.11	0.05	–0.11	–0.17	–0.09	0.04	0.12	0.03	0.15	0.18*	0.43**	(0.92)	
(15) Overall team performance	4.69	0.66	0.19*	–0.05	0.04	–0.03	0.03	–0.09	–0.04	0.06	0.08	–0.06	0.13	0.10	0.38**	0.38**	(0.91)

*n* = 130. * *p* < 0.05, ** *p* < 0.01; two-tailed test. The numbers in parentheses on the diagonal of the table are Cronbach’s alpha estimates.

Table 2 provides the summary of the MPM results for testing all of the hypotheses simultaneously. Specifically, Hypothesis 1a proposes a positive linkage between leader power sharing and team explorative learning. As shown in Model 1, power sharing was significantly positively associated with team explorative learning (β = 0.34, *p* < 0.001). Thus, Hypothesis 1a was supported. Hypothesis 1b predicates a positive relationship between leader management control and to team exploitative learning. As shown in Model 2, management control was positively related to team exploitative learning (β = 0.19, *p* < 0.01). Thus, Hypothesis 1b was supported. Hypothesis 2a proposes a positive association between team explorative learning and team creativity. Results in Model 3 showed that team explorative learning was positively related to team creativity (β = 0.23, *p* < 0.05), lending support to Hypothesis 2a. Hypothesis 2b identifies a positive relationship between team exploitative learning and team task completion. Results in Model 4 showed that team exploitative learning was positively associated with team task completion (β = 0.18, *p* < 0.05), providing support for Hypothesis 2b.

**TABLE 2:** Results for testing hypotheses.

	**Team explorative learning**			**Team exploitative learning**			**Team** **creativity**			**Team task completion**			**Overall team performance**		
	***B***	***SE***	**β**	***B***	***SE***	**β**	***B***	***SE***	**β**	***B***	***SE***	**β**	***B***	***SE***	**β**
					
	**Model 1**			**Model 2**			**Model 3**			**Model 4**			**Model 5**		
**Control variables:**															
Team tenure	–0.01	0.00	–0.06	0.01*	0.00	0.08*	−0.03*	0.01	−0.11*	0.02	0.01	0.09	−0.02***	0.01	−0.12***
Team size	0.00	0.01	0.03	–0.00	0.01	–0.03	–0.01	0.01	–0.10	–0.01	0.01	–0.10	–0.00	0.00	–0.04
R&D	0.24***	0.05	0.33***	−0.10*	0.04	−0.16*	0.80**	0.24	0.44***	0.03	0.18	0.02	0.21	0.14	0.17
Manufacture/operations	0.23***	0.04	0.21***	–0.09	0.07	–0.10	0.38*	0.18	0.14*	–0.03	0.20	–0.01	0.18	0.18	0.10
Marketing/sales	0.33***	0.08	0.31***	−0.14**	0.04	−0.16**	0.56	0.33	0.21	–0.14	0.18	–0.06	0.35***	0.09	0.20***
Customer service	0.20**	0.06	0.09**	0.34***	0.06	−0.17***	0.40	0.22	0.07	−0.62**	0.23	−0.13**	0.36*	0.14	0.10**
Functional management	0.05	0.11	0.03	–0.07	0.08	–0.05	–0.08	0.23	–0.02	–0.33	0.27	–0.09	0.27	0.31	0.09
Operation support	0.00	0.07	0.00	0.03	0.12	0.02	1.48***	0.37	0.26***	0.14	0.23	0.03	0.57	0.30	0.15*
**Predictors of interest:**															
Leader power sharing	0.30***	0.04	0.34***	0.14*	0.06	0.18*									
Leader management control	0.15*	0.06	0.15*	0.18**	0.06	0.19**									
Team explorative learning							0.58*	0.25	0.23*	0.17	0.22	0.08	–0.07	0.13	–0.04
Team exploitative learning							0.21	0.24	0.08	0.42*	0.18	0.18*	0.13	0.20	0.07
Team creativity													0.14*	0.06	0.21*
Team task completion													0.17*	0.08	0.21*
*R*^2^	0.211***			0.128***			0.195***			0.097*			0.137*		

*n* = 130. *B* represents unstandardized path coefficients; *SE* represents standard error; β represents standardized path coefficients. * *p* < 0.05, ** *p* < 0.01, *** *p* < 0.001; two-tailed test.

For the tests of mediation hypotheses (Hypothesis 3a and 3b), we used the Monte Carlo simulation to construct the confidence intervals of the focal indirect effects. Using 5000 resamples via R program, we found a significant positive indirect effect of team explorative learning on overall team performance via team creativity (*b* = 0.07, bias-corrected bootstrap 95% CI = [0.02, 0.34], excluding zero). Thus, Hypothesis 3a was supported. Similarly, we also found a significant positive indirect effect of team exploitative learning on overall team performance through team task completion (*b* = 0.08, bias-corrected bootstrap 95% CI = [0.01, 0.15], excluding zero). Thus, Hypothesis 3b was supported.

**FIGURE 2:**
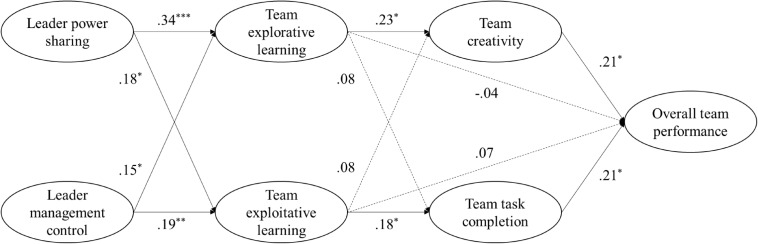
Overall path modeling results. Standardized path coefficients are reported. The dotted line represents non-significant results. *p < 0.05, **p < 0.01, ***p < 0.001.

In summary, our results provided support for all the hypotheses. The summary of these effects was presented in Figure 2.

### Exploratory Analyses

Surprisingly, we also found that leader power sharing is also positively related to team exploitative learning, and that leader management control is also positively related to team explorative learning. Theoretically, these findings inspire us to further considerate the antecedents of explorative and exploitative learning. Although team explorative learning is in demand of a more discretionary context (e.g., leader power sharing) that triggers variance of team processes, a certain level of constraint (e.g., leader management control) is useful because team members need some structuring and direction when exploring new things (Bain et al., 2001; Rosing et al., 2011). Similarly, despite team exploitative learning benefits more from a relative constrained context (e.g., leader management control) that reduces variance of team processes, it also requires necessary autonomy (e.g., leader power sharing) to explore new approaches to overcoming the unexpected challenges in the execution of tasks (Van de Ven, 1986; Rosing et al., 2011). Clearly, then, both leader power sharing and management control are useful for team explorative and exploitative learning, even if explorative learning is linked more closely to power sharing, while exploitative learning is more closely related to management control.

To empirically justify the above arguments, we used Mplus to statistically compare the difference between the path coefficients (i.e., the coefficient from leader power sharing to explorative learning vs the coefficient from leader management control to explorative learning; the coefficient from leader management control to exploitative learning vs. the coefficient from leader power sharing to exploitative learning). The results showed that the effect of leader power sharing on team explorative learning was significantly stronger than that of leader management control on team explorative learning (*difference score* = 0.15, *p* < 0.05), and that the effects of leader power sharing and management control on team exploitative learning had no significant difference (*difference score* = 0.03, *n.s.*). The plausible reason is that the participated teams are mainly R&D teams. In the process of executing plans or improving procedures (i.e., exploitative activities), these teams always face unexpected or ill-defined problems which requires necessary autonomy to seek for new solutions. In such condition, leader power sharing acts as an important driver of exploitative activities. We encourage future research to further test the effect difference on team exploitative learning between leader power sharing and management control by using samples from diverse functions.

## Discussion

The main purpose of this study was to establish a theoretical framework of team ambidextrous learning by examining the specific antecedents of team explorative and exploitative learning from the perspective of ambidextrous leader behaviors (i.e., power sharing and management control), and the specific mechanisms linking team explorative and exploitative learning to team effectiveness. In the following, we will discuss how our findings contribute to the existing literature and managerial practices, the limitations of this study, and future directions in the emerging field of team ambidextrous learning.

### Theoretical Implications

First, the results regarding the antecedents of team ambidextrous learning indicated that leader power sharing behavior positively predicted team explorative learning, and that leader management control behavior positively predicted team exploitative learning. These findings suggest that leaders who engage in loose–tight control behaviors influence their followers’ collective learning behaviors in ways that are consistent with leaders’ behaviors (i.e., delegating power or setting up constraints to team members). Although prior studies have elaborated ambidextrous leader behaviors (i.e., typically depicted as opening and closing leading behaviors) as an antecedent of employee ambidextrous behaviors (e.g., Rosing et al., 2011; Zacher et al., 2016), we have limited empirical evidence regarding what specific instruments are effective for team leaders to adopt to accelerate team explorative and exploitive activities respectively. Thus, drawing on the loose–tight model of leadership (Sagie, 1997), we specified the unique value of team leader’s empowering versus controlling behaviors in facilitating team explorative and exploitative learning. This attempt not only underscore the importance of considering leader influences when developing team ambidextrous learning capacity, but also enriches the research on the effectiveness of ambidextrous leader behaviors from the dimension of empowerment and control (Zhang et al., 2015). Considering that Kostopoulos and Bozionelos (2011) identified “team psychological safety” as an antecedent of team explorative and exploitative learning, and that leader behaviors are important to build safety climate (Edmondson, 2002), it’s interesting to explore whether team psychological safety serves as a more proximal engine to mediate the effects of leader empowering or controlling behaviors on team ambidextrous learning activities in the future research.

Second, our study adopts the multi-source data to validate the effectiveness of team ambidextrous learning by specifically examining the linkage between team explorative learning and team creativity, and that between team exploitative learning and team task completion. Although existing literature have confirmed that ambidexterity contributed to performance or innovation (e.g., Gibson and Birkinshaw, 2004; Zacher et al., 2016; Hunter et al., 2017; Ubeda-Garcia et al., 2019), few studies clarified the specific values of exploration and exploitation in driving performance or innovation, especially at the team level. Our results showed that team explorative learning (rather than exploitative learning) reported by team member was positively associated with leader-rated team creativity, and that team exploitative learning (rather than explorative learning) reported by team member was positively related to leader-rated team task completion. These findings provide a rigorous evidence supporting the specific value of ambidextrous activities in cultivating team change forces (i.e., creativity) or executive forces (i.e., task completion). In this way, we clearly differentiate team explorative and exploitative learning as separate and orthogonal subcomponents of the unitary construct of team ambidextrous learning.

Finally, we found that team explorative and exploitative learning positively predicated overall team performance through team creativity and task completion respectively. Extant literature primarily focused on the direct effects of team ambidextrous learning on team effectiveness, but provided limited evidence regarding the embedded mechanisms. As such, our findings add an additional value to the existing literature by identifying two mediators (i.e., team creativity and task completion) in the relationships between team explorative and exploitative learning and overall team performance. The two mediators represent the change-oriented and the execution-oriented mechanisms specifically, both of which are critical routes to achieving team effectiveness (Gilson et al., 2005). In addition, our study offers evidence supporting the premise of contextual ambidexterity which highlights explorative and exploitative learning can coexist to foster team effectiveness together, as the findings suggest that explorative and exploitative learning constitute two different sources of team overall performance via generating novel and useful ideas and finishing established tasks effectively. The most effective teams, therefore, should be those that are able to identify the type of situation they are experiencing and adjust their behavioral modes accordingly.

### Managerial Implications

Based on the findings, we have the following two managerial implications for practices. First, as market is increasingly competitive and dynamic, teams need to pursue equilibrium between the paradoxical demands. Our study demonstrated that the ambidextrous teams cannot only adapt to the changes of external environment by creating new knowledge, but also integrate existing knowledge to improve the internal efficiency through effective task completion. Therefore, as team managers, they need to know how to balance the tension between explorative and exploitative learning or that between creation and execution, and then build an ambidextrous team to access to the sustained competitive advantages.

In addition, prior studies indicate that leadership plays an important role in pursuing ambidexterity (e.g., O’Reilly and Tushman, 2013). Our research supports this point by showing how team leaders use power sharing and management control behaviors to specifically facilitate team explorative and exploitative learning. This provides an effective approach to be an ambidextrous leader for team managers. To develop ambidextrous leader behaviors, team managers need to learn how to shift their leading behaviors between empowering and controlling in accordance with the changes of focus between exploration and exploitation.

### Limitations and Future Directions

Our study also has limitations. First, although our measures of team ambidextrous learning were widely used in other team research (e.g., Kostopoulos and Bozionelos, 2011; Jansen et al., 2016), we should acknowledge that these measures only capture the processes of learning (i.e., what is team ambidextrous learning) while ignoring the information about how to balance the tension between exploration and exploitation (i.e., how to achieve the state of team ambidexterity). Considering the original definition of ambidextrous leadership proposed by Rosing et al. (2011) included a dimension of temporal flexibility to switch leading behaviors, we suggest that future research should employ other methods (e.g., case study) to unpack how leaders shift their focus between empowering- and controlling-oriented leading behaviors based on the changes of context to achieve the dynamic equilibrium between explorative and exploitative activities. By doing so, we can get a better answer to the question that how to build an ambidextrous team from the leadership perspective.

Second, as our data were solely collected in China which is a country with relatively high levels of power distance, some cultural issues may be embedded in our findings. Prior studies suggest that cultural differences impact employees’ interpretation of their leaders’ behaviors (Hofstede, 1980). For example, in a high power-distance culture, employees are more willingness to be obedience to leaders’ authority (e.g., Tyler et al., 2000; Farh et al., 2007; Humborstad et al., 2008). Thus, in our empirical context, team leaders’ management control rather than power sharing behaviors may have a more significant impact on team members’ collective activities. Based on this, we encourage future research to replicate our findings in other cultural backgrounds.

Finally, although we adopted a time-lagged and multisource design to avoid the potential common-method bias issue (Podsakoff et al., 2012), we cannot draw on causal conclusions in terms of our theoretical model. For example, an ambidextrous leader may select the members who also have ambidextrous capacities to build up his/her team, and tend to amplify their contributions in the process of performance appraisal. Therefore, we suggest that researchers should employ a repeated-measure-based longitudinal or experimental design to further justify the causal relationships in our hypotheses.

## Conclusion

Considering that contemporary fierce competition requires teams to develop new products or services to satisfy customers’ changing needs while refining existing products or services to implement quality improvement (Gilson et al., 2005), our study investigates how teams can be ambidextrous to integrate the opposing demands from the perspective of ambidextrous leader behaviors. Furthermore, our study advances research on the effectiveness of team ambidextrous learning by showing that team explorative and exploitative learning enhance overall team performance through the change-oriented mechanism (i.e., team creativity) and the execution-oriented mechanism (i.e., task completion), respectively. We hope that our theoretical model and empirical evidences will stimulate more research attention on how teams can facilitate the pursuit of ambidexterity.

## Data Availability Statement

The datasets generated for this study are available on request to the corresponding author.

## Author Contributions

KZ and BZ were both involved in conceptualizing the study, the data analysis, and writing the manuscript. KZ was responsible for the data collection. LZ provided comments and suggestions to further elaborate the manuscript. All authors contributed to the article and approved the submitted version.

## Conflict of Interest

The authors declare that the research was conducted in the absence of any commercial or financial relationships that could be construed as a potential conflict of interest.

## Appendix 1

### Scale Items

(1) **Leader Power Sharing Scales** (1 strongly disagree to 6 strongly agree)

 (1) My supervisor does not interfere with work that is within my job responsibility.
   (2) My supervisor fully delegates and lets me take full charge of my job.
   (3) My supervisor gives me authority to make autonomous decisions in my job.
   (4) When there is a problem at work, my supervisor listens to my ideas and suggestions.
   (5) My supervisor often provides me opportunities to express my ideas.
   (6) My supervisor asks for my views before making decisions concerning me and my job.
   (7) When making decisions, my supervisor respects and values my suggestions.
   (2) **Leader Management Control Scales** (1 strongly disagree to 6 strongly agree)
   (1) My supervisor sets work goal for me and requires me to ensure its achievement.
   (2) My supervisor emphasizes work goals.
   (3) My supervisor periodically checks if I am accomplishing my work goals.
   (4) My supervisor emphasizes work outcome.
   (5) My supervisor would seriously point out my work mistakes.
   (6) My supervisor often asks me about my work progress.
   (7) My supervisor periodically examines whether my work is going on smoothly.
   **(3) Team Explorative Learning Scales** (1 strongly disagree to 6 strongly agree)
   (1) Team members were systematically searching for new possibilities during the project.
   (2) Team members offered new ideas and solutions to complicated problems (were inventive).
   (3) Team members experimented with new and creative ways for accomplishing work.
   (4) Team members evaluated diverse options regarding the course of the project.
   (5) The members of our team developed many new skills during the project.
   (4) **Team Exploitative Learning Scales** (1 strongly disagree to 6 strongly agree)
   (1) The members of our team recombined existing knowledge for accomplishing work.
   (2) In our team, we primarily performed routine activities.
   (3) During the project, our team implemented standardized methodologies and regular work practices.
   (4) Team members improved and refined their existing knowledge and expertise during the project.
   (5) Team members mainly used their current knowledge and skills for performing their tasks.
   (5) **Team Creativity Scales** (1 poorly to 6 very much)
   (1) How well does your team produce new ideas?
   (2) How useful are those ideas?
   (3) How significant are those ideas?
   (4) How creative do you consider your team to be?
   **(6) Team Task Completion Scales** (1 strongly disagree to 6 strongly agree)
   (1) This team achieves its goals.
   (2) This team accomplishes its objectives.
   (3) This team meets the requirements set for it.
   (4) This team fulfills its mission.
   (5) This team serves the purpose it is intended to serve.
   **(7) Overall Team Performance** (1 far below average to 6 far above average)
   (1) Efficiency
   (2) Quality
   (3) Overall achievement
   (4) Productivity
   (5) Mission fulfillment

## Appendix 2

### Within-Group Agreement Statistics

**Table:**

	**Rwg (uniform)**	**Rwg (measure-specific)**		
**Measures**	**Mean**	**Shape**	**σ^2^_E_**	**Mean**
Leader power sharing	0.84	Moderate skew	1.26	0.45
Management control	0.87	Normal	1.45	0.48
Team explorative learning	0.82	Slight skew	1.85	0.28
Team exploitative learning	0.88	Moderate skew	1.26	0.33

*Shape = the shape of an alternative null distribution; σ^2^_*E*_ = variance of an alternative null distribution. Variance estimations for measure-specific null distributions (i.e., slight skew, normal, and moderate skew).*
